# Extensive shared polymorphism at non-MHC immune genes in recently diverged North American prairie grouse

**DOI:** 10.1007/s00251-017-1024-4

**Published:** 2017-08-02

**Authors:** Piotr Minias, Zachary W. Bateson, Linda A. Whittingham, Jeff A. Johnson, Sara Oyler-McCance, Peter O. Dunn

**Affiliations:** 1 Department of Biodiversity Studies and Bioeducation, Faculty of Biology and Environmental Protection, University of Łódź, Banacha 1/3, 90-237 Łódź, Poland; 2 Behavioral and Molecular Ecology Group, Department of Biological Sciences, University of Wisconsin-Milwaukee, Milwaukee, WI USA; 3 Department of Biological Sciences, Institute of Applied Sciences, University of North Texas, Denton, TX USA; 4 Fort Collins Science Center, US Geological Survey, Ft. Collins, CO USA

**Keywords:** Ancestral polymorphism, Grouse, Immune genes, Introgression, Reciprocal monophyly, Selection

## Abstract

Gene polymorphisms shared between recently diverged species are thought to be widespread and most commonly reflect introgression from hybridization or retention of ancestral polymorphism through incomplete lineage sorting. Shared genetic diversity resulting from incomplete lineage sorting is usually maintained for a relatively short period of time, but under strong balancing selection it may persist for millions of years beyond species divergence (balanced trans-species polymorphism), as in the case of the major histocompatibility complex (MHC) genes. However, balancing selection is much less likely to act on non-MHC immune genes. The aim of this study was to investigate the patterns of shared polymorphism and selection at non-MHC immune genes in five grouse species from *Centrocercus* and *Tympanuchus* genera. For this purpose, we genotyped five non-MHC immune genes that do not interact directly with pathogens, but are involved in signaling and regulate immune cell growth. In contrast to previous studies with MHC, we found no evidence for balancing selection or balanced trans-species polymorphism among the non-MHC immune genes. No haplotypes were shared between genera and in most cases more similar allelic variants sorted by genus. Between species within genera, however, we found extensive shared polymorphism, which was most likely attributable to introgression or incomplete lineage sorting following recent divergence and large ancestral effective population size (i.e., weak genetic drift). Our study suggests that North American prairie grouse may have attained relatively low degree of reciprocal monophyly at nuclear loci and reinforces the rarity of balancing selection in non-MHC immune genes.

**Electronic supplementary material:**

The online version of this article (doi:10.1007/s00251-017-1024-4) contains supplementary material, which is available to authorized users.

## Introduction

Genetic variation shared between recently diverged taxa is most commonly explained with retention of ancestral polymorphisms or introgressive hybridization (Donnelly et al. 2004; Zhou et al. 2017). The first mechanism is consistent with incomplete lineage sorting when ancestral allelic variants are maintained in both descendant species following neutral expectation. Incomplete lineage sorting is widespread and likely to persist for some period of time after species divergence, depending on the strength of drift (negatively correlated with the effective population size) and the mutation rate for a particular set of loci (Hudson and Coyne 2002; Hedrick 2013). Similar patterns of shared genetic diversity are also produced by introgression, i.e., the horizontal transfer of allelic lineages, which may either contribute to the adaptive genetic variation of the recipient species or may be non-adaptive (detrimental or neutral). Recently, it has been suggested that introgression may be much more common in vertebrates than previously thought (Rheindt and Edwards 2011; Hedrick 2013), and there is accumulating evidence for the role of introgression in generating variation at immune genes of different animal taxa (Parmakelis et al. 2008; Nadachowska-Brzyska et al. 2012; Grossen et al. 2014).

Assuming that multiple ancestral allelic variants are adaptive, their polymorphism shared by descendant species may be maintained by balancing selection for longer periods of time beyond neutral expectations, which is often referred to as balanced trans-species polymorphism (TSP). However, shared polymorphism can also evolve independently in two or more lineages from different ancestral states as a result of adaptation to similar selective pressures (convergent evolution). Among the immune genes, balanced TSP and convergent evolution have been almost exclusively reported for the major histocompatibility complex (MHC), where balancing selection can maintain ancestral allelic variants for millions of years following divergence (Aguilar and Garza 2007; Kamath and Getz 2011) and exposure to similar pathogen pressure can produce convergence at the amino acid sequence level (Yeager et al. 1997; Kriener et al. 2000; Srithayakumar et al. 2012). So far, molecular evidence for balanced TSP and convergence at non-MHC immune genes are very limited (Leulier and Lemaitre 2008; Téšický and Vinkler 2015).

The aim of this study was to investigate the patterns of shared polymorphism at non-MHC immune genes in five grouse species from two closely-related genera, *Centrocercus* and *Tympanuchus* (Drovetski 2003; Galla and Johnson 2015; Stein et al. 2015). We examined five immune genes that do not directly interact with pathogens, but are involved in signaling and regulating immune cell growth: chicken B cell marker 6 (ChB6 or Bu-1), inhibitor of apoptosis protein-1 (IAP-1), interleukin-2 (IL-2), transforming growth factor β3 (TGF-β3), and tumor necrosis factor-related apoptosis inducing ligand-like protein (TRAIL-like). Polymorphisms at these genes were associated with general mortality and growth (Ye et al. 2006), as well as with specific fitness-related immune traits in a close relative of grouse, the domestic chicken *Gallus gallus*. Polymorphisms at ChB6 were associated with regression of Rous sarcoma and resistance to Marek’s disease (Gilmour et al. 1986; Taylor et al. 2016), while IAP-1 polymorphism showed associations with *Salmonella enteritidis* burden in the spleen (Liu and Lamont 2003) and antibody response kinetics to *Brucella abortus* (Zhou and Lamont 2003). Fitness associations for three of these genes have been reported for synonymous SNPs and SNPs in non-translated genic regions, suggesting their linkage to functional polymorphisms. For example, a synonymous and an intronic polymorphism at TRAIL-like and TGF-β3 genes, respectively, were associated with bacterial load in the cecum and spleen contents of chickens (Malek and Lamont 2003).

Based on fossil-calibrated gene trees using both nuclear and mtDNA loci, the divergence between *Centrocercus* and *Tympanuchus* is estimated at ca. 6 to 8 million years, and species divergence within the two genera was within the last million years (Galla and Johnson 2015; Stein et al. 2015; Persons et al. 2016). While no information exists on the occurrence of *Centrocercus* hybrids (Young et al. 2015), hybridization has been widely documented between *Tympanuchus* species in areas of geographic overlap (Bain and Farley 2002; Augustine and Trauba 2015; Oyler-McCance et al. 2016) and hybrids appear to persist across generations due to sex-biased introgression (Galla and Johnson 2015). In contrast, *Centrocercus*-*Tympanuchus* hybrids are rare, and back-crossing is unlikely due to behavioral post-zygotic isolation (Aldridge et al. 2001; see also Augustine and Trauba 2015). Thus, we expected extensive shared polymorphisms within each of the two grouse genera, *Tympanuchus* and *Centrocercus*, resulting from incomplete lineage sorting and/or introgression and low or absent levels of shared polymorphism between genera due to accumulating mutations and drift.

## Material and methods

### Sample collection

**Fig. 1:**
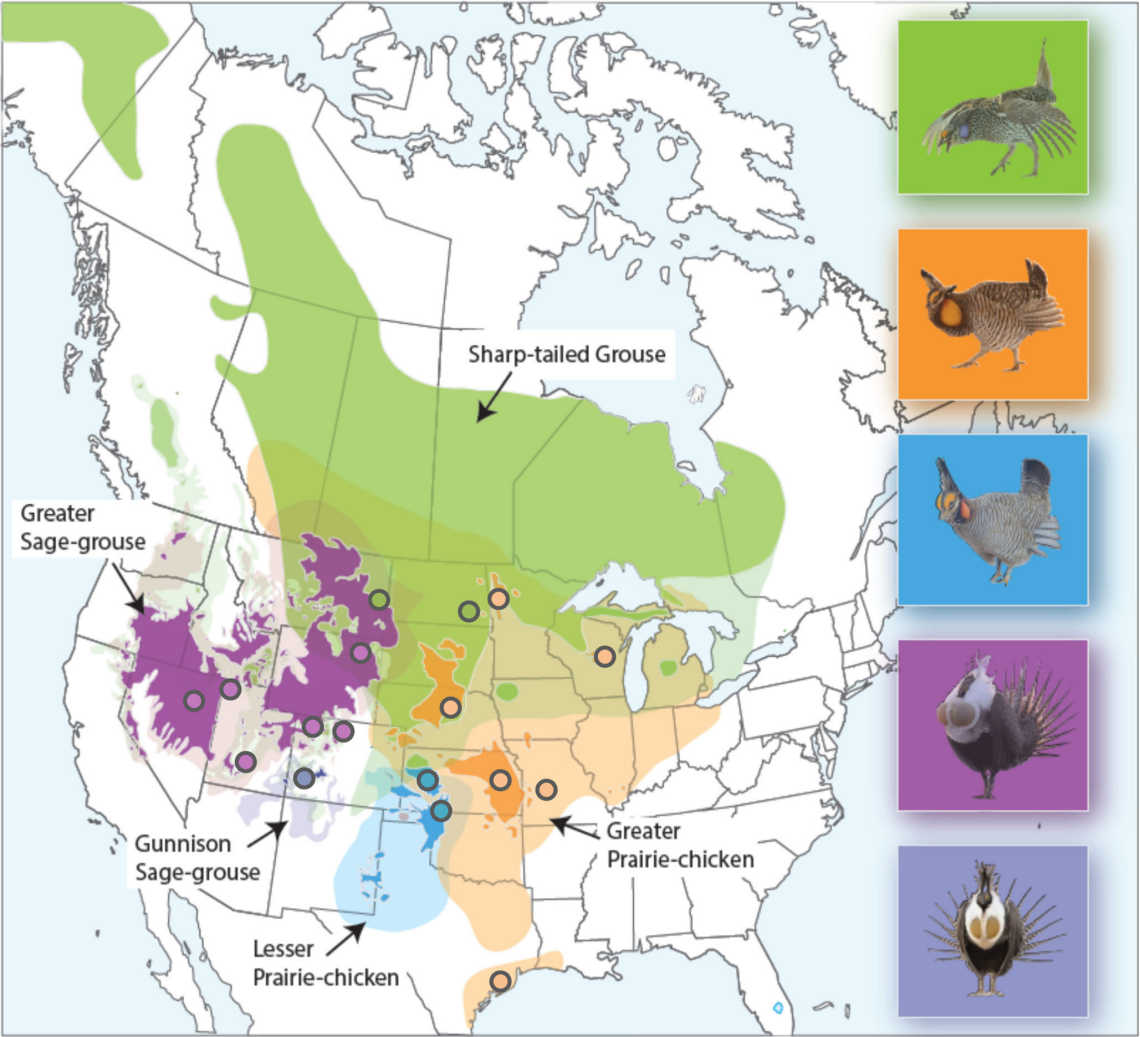
Contemporary (*full color*) and historic (*semi-transparent color*) distributions of *Centrocercus* (Gunnison sage-grouse *C. minimus*—*dark blue*, greater sage-grouse *C. urophasianus—violet*) and *Tympanuchus* (greater prairie-chicken *T. cupido*—*orange*, lesser prairie-chicken *T. pallidicinctus*—*light blue*, sharp-tailed chicken *T. phasianellus*—*green*) grouse. Approximate locations of sampled populations are shown

Samples were collected for five species of North American grouse: *Centrocercus minimus* (Gunnison sage-grouse), *C. urophasianus* (greater sage-grouse), *Tympanuchus phasianellus* (sharp-tailed grouse), *Tympanuchus pallidicinctus* (lesser prairie-chicken), and *Tympanuchus cupido* (greater prairie-chicken). These species are the only members of *Centrocercus* and *Tympanuchus* genera and together with two *Dendragapus* species form a monophyletic clade within Tetraoninae (Drovetski 2003; Stein et al. 2015). Blood and feather samples for the greater prairie-chicken were collected between 1990 and 2006 from six populations (Kansas, Minnesota, Missouri, Nebraska, Texas, and Wisconsin; Fig. 1). In total, 247 individuals were sampled and previously genotyped at all five immune genes by Bollmer et al. (2011). For the remaining species, blood and tissue samples (30 individuals per species) were collected between 1997 and 2011 in the following states: Colorado (*C. minimus, C. urophasianus*), Nevada and Utah (*C. urophasianus*), Wyoming (*C. urophasianus, T. phasianellus*), North Dakota (*T. phasianellus*), and Kansas (*T. pallidicinctus*) (Fig. 1). Protocols used for DNA extraction are described elsewhere (Bellinger et al. 2003; Oyler-McCance et al. 2005; Galla and Johnson 2015).

### Laboratory analyses

We examined five non-MHC immune genes, which do not interact directly with pathogens (ChB6, IAP-1, IL-2, TGF-β3, and TRAIL-like). ChB6 is a B cell surface antigen that induces a physiological signal for apoptosis in self-reactive lymphocytes, thus preventing autoimmune diseases in birds (Funk et al. 2003). IAP-1 is involved in antiapoptotic pathways including binding and inhibiting caspases and modulating receptor-mediated signal transduction (Yang and Li 2000). IL-2 plays a critical role in immune system function, as it induces the proliferation and differentiation of T, B, and natural killer (NK) cells (Zhou et al. 2001). TGF-β3 belongs to a large family of cytokines which are cell-cell signaling proteins involved in the processes of proliferation, differentiation, growth, and migration of immune cells (Elliott and Blobe 2005). TRAIL-like cytokine stimulates apoptotic cell death and activates cytotoxic T cells, playing a role in the clearance of tumors and some viral infections (Falschlehner et al. 2009).

Amplifications of all five genes were performed using primer sets and annealing temperatures published for the domestic chicken (ChB6 and IAP-1, Zhou and Lamont 2003; IL-2, Zhou et al. 2001; TGF-β3, Ye et al. 2006; TRAIL-like, Malek and Lamont 2003), and previously tested for the greater prairie-chicken (Bollmer et al. 2011). The following regions of the genes were amplified: (1) ChB6: exon encoding the extracellular part of the molecule; (2) IAP-1: exon encoding the BIR motif essential for gene function; (3) IL-2: promoter with a fragment of exon 1; (4) TGF-β3: intron 4 with a fragment of exon 5; and (5) TRAIL-like: intron 1 with a fragment of exon 1 (Table 1). The exon fragments amplified in the IL-2, TGFβ3, and TRAIL-like genes were small (55–103 bp) with little sequence variation (0–1 SNP); therefore, we discarded these parts of the sequences and focused our analyses on the promoter (IL-2) and intron (TGFβ3 and TRAIL-like) regions. Analyses of diversity at exons were confined to ChB6 and IAP-1. All genes were amplified in 20-μl polymerase chain reactions (PCRs) following methods described elsewhere (Bollmer et al. 2011). All PCR products were sequenced using Sanger sequencing technology in both forward and reverse directions at the University of Chicago Cancer Research Center DNA Sequencing Facility. Sequences not included among previously identified alleles (accession nos.: JN573105–JN573162) were deposited in GenBank (accession nos.: MF579259-MF579341). Each amplified gene region was considered orthologous, as haplotypes showed high nucleotide identity across all five grouse species (> 95% identity), as well as with haplotypes derived from other galliform species (domestic chicken, Japanese quail *Coturnix japonica*, and turkey *Meleagris gallopavo*; > 90% identity). There was no evidence for copy number variation of these genes in this or previous studies on the domestic chicken (Zhou et al. 2001, Malek and Lamont 2003, Zhou and Lamont 2003, Ye et al. 2006) and greater prairie-chicken (Bollmer et al. 2011).

**Table 1:** Polymorphism of five immune genes in *Centrocercus* and *Tympanuchus* grouse

	ChB6	IAP-1	IL-2	TGF-β3	TRAIL-like
Fragment region	Exon 3	Exon 2	Promoter	Intron 4	Intron 1
Fragment size	114 bp	323 bp	412 bp	685 bp	555 bp
No. of variable sites	6	7	14	24	34
No. of haplotypes	11	8	15	19	35
Average no. of nucleotide differences	2.62 ± 0.18	1.93 ± 0.35	2.19 ± 0.21	4.80 ± 0.63	4.53 ± 0.30

### Analysis of diversity and selection

All sequences were assembled, edited, and aligned in Geneious v7.1.7 (Biomatters Ltd., Auckland New Zealand). Sequences from each gene were assigned to haplotypes separately for each species using the PHASE algorithm (Stephens and Donnelly 2003) in DnaSP v5.0 (Librado and Rozas 2009). We used a burn-in of 1000 iterations, followed by 1000 iterations and a thinning interval of ten. Sequence polymorphism (total number of mutations and average number of nucleotide differences) across all five grouse species was analyzed with DnaSP v5.0.

We used MEGA v.6.0 (Tamura et al. 2013) to compare the fit of three basic nucleotide substitution models with variable base frequencies: (1) Hasegawa-Kishino-Yano (HKY) model, which assumes one transition rate and one transversion rate (Hasegawa et al. 1985); (2) Tamura-Nei (TN) model, which assumes variable transition rates and equal transversion rates (Tamura and Nei 1993); and (3) General Time Reversible (GTR) model, which assumes a symmetrical substitution matrix (Tavaré 1986). For both exon fragments, the HKY model showed the best fit, as indicated by the lowest corrected Akaike’s Information Criterion (TN: ΔAIC_C_ = 4.37 for ChB6, ΔAIC_C_ = 1.31 for IAP-1; GTR: ΔAIC_C_ = 11.33 for ChB6, ΔAIC_C_ = 4.62 for IAP-1). Thus we used the HKY model in all further analyses. To test for positive and purifying selection on ChB6 and IAP-1 exon fragments, we assessed the relative rates of non-synonymous (dN) and synonymous (dS) nucleotide substitutions across the sequences (Hughes and Nei 1988). In general, a positive value for dN-dS indicates an overabundance of nonsynonymous substitutions, which is consistent with positive selection, while the negative value indicates purifying selection. Codon-specific signatures of positive and purifying selection on ChB6 and IAP-1 exon fragments were tested using three different approaches implemented in the Datamonkey web server (Delport et al. 2010): single likelihood ancestor counting (SLAC), fixed effects likelihood (FEL), and random effects likelihood (REL). SLAC is considered to be the most conservative approach, while REL has been reported to suffer from higher rates of false positives for small data sets (Kosakovsky Pond & Frost 2005). Under most scenarios, FEL approach is intermediate between SLAC and REL in terms of type I error (Kosakovsky Pond & Frost 2005). We also used mixed effects model of evolution (MEME) to detect episodic diversifying selection affecting individual codon sites (Murrell et al. 2012).

### Phylogenetic analyses and haplotype networks

Phylogenetic analyses were conducted using Bayesian inference (Markov Chain Monte Carlo method, MCMC) as implemented in MrBayes 3.2.6 (Huelsenbeck and Ronquist 2001). We specified the HKY model of nucleotide substitution. Four chains were set to run for 500,000 generations with a burn-in length of 100,000. Trees were sampled every 100 generations for a total of 5000 trees. The initial 1000 sampled trees were discarded as the burn-in, and the 50% majority-rule Bayesian consensus trees and associated clade posterior probabilities were computed from the remaining 4000 trees. The domestic chicken *Gallus gallus* was used as the outgroup (GenBank accession nos.: X92865, ChB6; AF008592, IAP-1; AJ224516, IL-2; X60091, TGF-β3; and AF537189, TRAIL-like). To further visualize differentiation of each gene and frequencies of phased haplotypes, we constructed median-joining haplotype networks (Bandelt et al. 1999) using the program Network v.4.6.10 (available at: www.fluxus-engineering.com).

## Results

### Diversity and selection

Each gene for all five grouse species had between six and 34 variable sites (Table 1), with a total of 85 variable nucleotide sites for all five immune genes. Most of the variable sites (68%) were found within introns (TGF-β3 and TRAIL-like). None of the variable sites were shared between *Centrocercus* and *Tympanuchus* genera (Fig. S1 in Electronic Supplementary Material). Haplotype reconstruction indicated the presence of 8 to 35 haplotypes per immune gene (Table 1), with a higher number of haplotypes observed for intron sequences (61%). Introns were also associated with higher average nucleotide differences than the exons or the promoter sequences (Table 1). Among all five grouse species, *C. minimus* had the fewest haplotypes, both for exon and non-exon sequences (*n* = 7 haplotypes in total), and both *Centrocercus* species had fewer haplotypes than observed in the *Tympanuchus* species (Table 2). *T. cupido* had the highest number of haplotypes (Table 2), but this was likely due to a much larger sample size (*n* = 247) when compared to each of the remaining species (*n* = 28–30, Table 2).

**Table 2:** Within-species polymorphism of five immune genes in *Centrocercus* and *Tympanuchus* grouse, where *n*_IND_ is the number of individuals genotyped, *n*_VS_ is the number of variable sites and *n*_HAP_ is the number of SNP haplotypes (number of private haplotypes *n*_PRIV.HAP_ in parentheses)

Genus/species	*n*_IND_	*n*_VS_/*n*_HAP_ (*n*_PRIV.HAP_)					
		ChB6	IAP-1	IL-2	TGF-β3	TRAIL-like	Total
*Centrocercus*							
*C. minimus* (Gunnison sage-grouse)	30	0/1 (0)	0/1 (0)	1/2 (1)	0/1 (0)	3/2 (0)	4/7 (1)
*C. urophasianus* (greater sage-grouse)	29–30	2/4 (3)	1/2 (1)	0/1 (0)	1/2 (1)	8/7 (5)	13/17 (10)
*Tympanuchus*							
*T. phasianellus* (sharp-tailed grouse)	28–30	3/4 (0)	3/4 (0)	5/5 (4)	7/9 (9)	7/9 (3)	25/31 (16)
*T. pallidicinctus* (lesser prairie-chicken)	30	3/4 (0)	2/3 (0)	5/6 (2)	1/2 (1)	5/6 (1)	16/21 (4)
*T. cupido* (greater prairie-chicken)	247	4/7 (3)	5/6 (2)	6/7 (3)	7/7 (6)	20/24 (14)	42/51 (28)

We found little evidence for positive selection acting on ChB6 and IAP-1 genes. For ChB6, a signature of positive selection was detected for one codon (no. 13; Fig. S1 in ESM) using the REL method (*p* = 0.01), but this result was not confirmed with more conservative approaches (FEL: *p* = 0.25, SLAC: *p* = 0.28). No evidence for positive selection was found for the codons of the IAP-1 exon (all *p* > 0.05). Purifying selection was detected with REL and FEL approaches for one ChB6 codon (no. 18; REL: *p* < 0.01, FEL: *p* = 0.01; Fig. S1 in ESM), but not for any of the IAP-1 codons (all *p* > 0.05). Also, none of ChB6 and IAP-1 codons showed signatures of episodic diversifying selection with a MEME analysis (all *p* > 0.05).

### Allele sharing and phylogenetic analyses

**Fig. 2:**
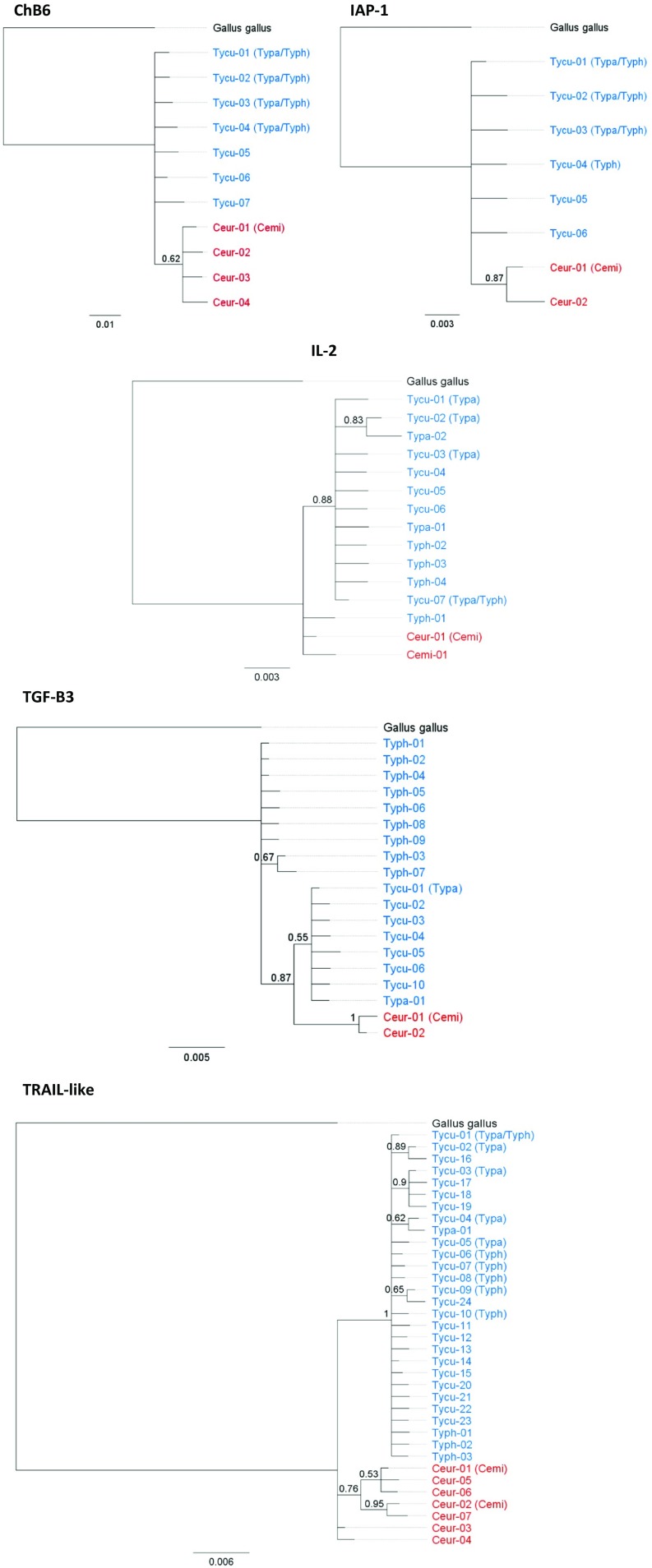
Bayesian 50% majority-rule consensus trees obtained for five immune genes (exons: ChB6 and IAP-1; promoter: IL-2; introns: TGF-β3 and TRAIL-like) in *Centrocercus* (*Cemi C. minimus*, *Ceur C. urophasianus*) and *Tympanuchus* (*Tycu T. cupido*, *Typa T. pallidicinctus*, *Typh T. phasianellus*) grouse. Alleles shared between species are indicated by acronyms given in *brackets*. *Centrocercus* and *Tympanuchus* alleles are marked in *red* and *blue*, respectively. Weakly supported nodes are represented as unresolved. Bayesian posterior probabilities above 50% are indicated at the nodes. The domestic chicken *Gallus gallus* was used as the outgroup

No haplotypes were shared between *Centrocercus* and *Tympanuchus* genera. In contrast, there was a high rate of exon haplotype sharing among species within genera. Sixty-two percent of *T. cupido* exon haplotypes (ChB6 and IAP-1; *n* = 13) were shared with other *Tympanuchus* species, and approximately half of those haplotypes (*n* = 7) were shared among all three *Tympanuchus* species. No private exon haplotypes were found in *T. phasianellus* or *T. pallidicinctus* (Table 2). The two *C. minimus* exon haplotypes were shared with *C. urophasianus* (Fig. 2).

Sharing of non-exon haplotypes was lower than with the exon haplotypes. Thirty-nine percent of *T. cupido* non-exon haplotypes (IL-2, TGF-β3, and TRAIL-like; *n* = 38) were shared with other *Tympanuchus* species and only two non-exon haplotypes (3%) were shared among all three *Tympanuchus* species (Fig. 2). A total of 70 and 29% of non-exon haplotypes were private in *T. phasianellus* (*n* = 23) and *T. pallidicinctus* (*n* = 14), respectively (Table 2). Four out of the five *C. minimus* non-exon haplotypes were shared with *C. urophasianus*.

**Fig. 3:**
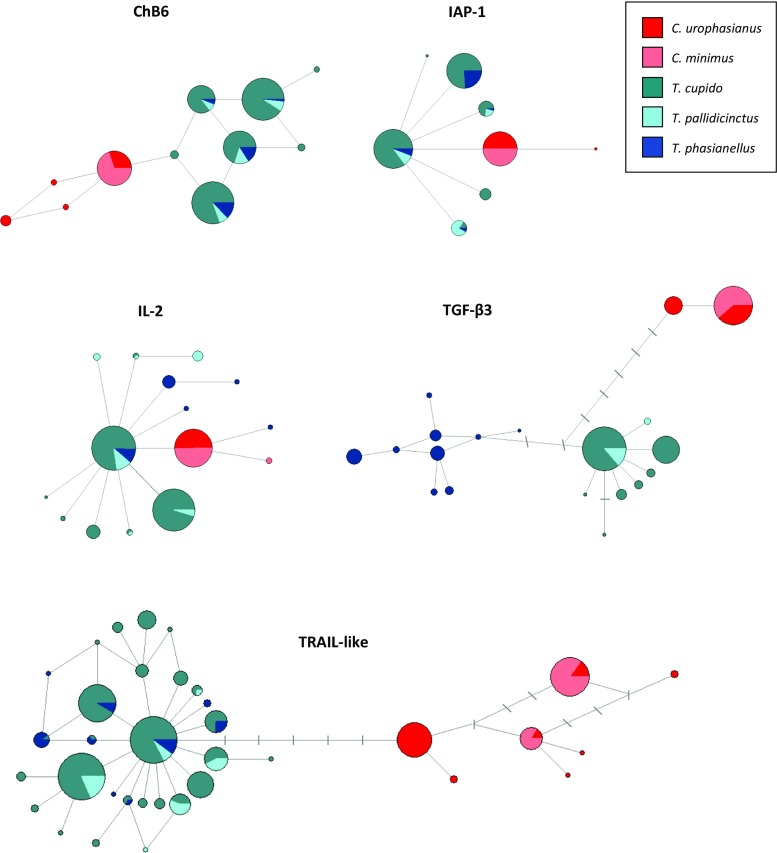
Median-joining haplotype networks for five immune genes (exons: ChB6 and IAP-1; promoter: IL-2; introns: TGF-β3 and TRAIL-like) in *Centrocercus* and *Tympanuchus* grouse. *Circles* represent individual haplotypes, and the diameter of each circle corresponds to the number of samples with that particular haplotype. *Tick marks* between circles denote mutational steps required to connect haplotypes

Four out of the five genes clustered by genus, as either *Centrocercus* or *Tympanuchus* haplotypes formed separate monophyletic clades (Figs. 2 and 3). The only exception was the IL-2 promoter gene, where two *Centrocercus* haplotypes formed a polytomy with one private *T. phasianellus* haplotype (Figs. 2 and 3). For the three genes (ChB6, IAP-1, and TGF-β3) monophyletic *Centrocercus* clades had moderate or high (0.62–1.00) Bayesian posterior probabilities (Fig. 2), while for the TRAIL-like gene, *Tympanuchus* haplotypes formed a monophyletic clade with 100% Bayesian posterior probability (Fig. 2). For the ChB6 and IAP-1 exons, *Centrocercus* and *Tympanuchus* clades were connected by only one mutational step, while for the TGF-β3 and TRAIL-like introns, *Centrocercus* and *Tympanuchus* clades were several mutational steps apart (Fig. 3). In most genes (except for TGF-β3), *Tympanuchus* haplotypes did not sort by species (Figs. 2 and 3). In contrast, all TGF-β3 haplotypes of *T. phasianellus* formed a clade separated by three mutational steps from other *Tympanuchus* haplotypes (Figs. 2 and 3), supporting an earlier divergence of this species from the lineage leading to *T. cupido* and *T. pallidicinctus*.

## Discussion

Our study revealed that several non-MHC immune genes showed extensive shared polymorphism within each of the two grouse genera, *Centrocercus* and *Tympanuchus*. At exons of ChB6 and IAP-1, all allelic variants in *T. phasianellus* and *T. pallidicinctus* were shared with *T. cupido*, and a similar pattern was found within *Centrocercus*, in which all alleles of *C. minimus* were shared with *C. urophasianus*. While the rate of allele sharing for promoter (IL-2) and intron (TGF-β3 and TRAIL-like) gene regions was lower, we did find several cases of non-exon allelic variants shared between species within genera (Fig. 2). In contrast, we found no evidence for allele sharing between genera. Allelic variants mostly sorted by genus, showing that ancestral polymorphism was not maintained following the divergence of *Centrocercus* and *Tympanuchus* genera.

Extensive shared polymorphism within *Centrocercus* and *Tympanuchus* grouse can be most likely explained by incomplete lineage sorting or introgression from hybridization. Species within each genus are thought to have diverged recently (within the last million years; Galla and Johnson 2015; Stein et al. 2015) and, thus, ancestral allelic lineages might have been retained in the descendant taxa, as there was not enough time for mutations to accumulate and achieve reciprocal monophyly. In most cases, shared genetic diversity resulting from incomplete lineage sorting is maintained for a relatively short period of time after species divergence. Under a simple allopatric speciation model, it takes roughly 9–12 *N*_*e*_ (historical effective population size) generations to make incipient species reciprocally monophyletic at more than 95% of neutral nuclear loci (Hudson and Coyne 2002). Loci under directional selection are likely to require even shorter time to attain reciprocal monophyly, unless selection is counterbalanced by any retarding effects of population structure (Hudson and Coyne 2002). Finally, mitochondrial DNA becomes monophyletic more rapidly than a single nuclear gene, and far more rapidly than a sample of several nuclear genes. mtDNA also has greater potential for becoming monophyletic by selective sweeps and, thus, caution should be exercised when using mtDNA to infer genome-wide reciprocal monophyly in recently diverged taxa (Hudson and Coyne 2002). Despite this, molecular evidence for incomplete lineage sorting based on nuclear loci is relatively rare in birds (Rheindt et al. 2009; Qu et al. 2012).

The effects of incomplete lineage sorting are notoriously difficult to distinguish from recurrent gene flow, as both scenarios produce a similar pattern of allele sharing (Holder et al. 2001; Hedrick 2013). In our study system, intra-generic hybridization might be common in sympatric populations of grouse (Augustine and Trauba 2015), and female backcrosses may well result in introgression (Galla and Johnson 2015). Considering the limitations of our data (few nuclear loci genotyped), incomplete lineage sorting and introgression represent equally viable explanations for extensive shared polymorphisms found within *Centrocercus* and *Tympanuchus* grouse genera. In fact, molecular studies of birds have often suggested that the two mechanisms may operate at the same time in recently diverged populations or species (e.g., Milá et al. 2007; Campagna et al. 2010; Qu et al. 2012).

We found no evidence for shared polymorphism of the five non-MHC immune genes between *Centrocercus* and *Tympanuchus* grouse genera. In most cases, allelic variants sorted by genus, indicating that ancestral polymorphism was not maintained for long evolutionary periods in these taxa. Since none of the variable sites were shared between the two genera, there was no signature of convergent evolution within these immune genes. Also, we found no evidence for episodic diversifying selection acting on the ChB6 and IAP-1 exons, and no support for an excess of non-synonymous versus synonymous nucleotide substitutions in these sequences (as measured with dN and dS), which could be indicative for balancing selection (Hughes and Nei 1988). These results are contrary to the patterns found at the MHC of *Centrocercus* and *Tympanuchus* grouse. There was evidence for allele sharing between the two grouse genera at both MHC classes I and II, although the strength of TSP was much weaker at MHC class I (Minias et al. 2016). This was consistent with contrasting patterns of selection at the two MHC classes in grouse, where class I was subject to much weaker diversifying and balancing selection (Minias et al. 2016). Although balancing selection may preserve MHC polymorphism for millions of years beyond species divergence (Ottová et al. 2005; Cutrera and Lacey 2007; Kamath and Getz 2011), the occurrence of balanced TSP at non-MHC immune genes seems to be scarce. A recent review by Téšický and Vinkler (2015) reported only a few non-MHC immune loci where shared polymorphisms could have been maintained by balancing selection. Some of the most notable examples include the oligoadenylate synthesase gene, which shows two deeply diverged allelic lineages predating the split of the house mouse *Mus musculus* and servant mouse *Mus famulus* 2.8 million years ago (Ferguson et al. 2008), and the immunoglobulin IgA hinge region, which shows balanced TSP in primates (Sumiyama et al. 2002).

In conclusion, our study provided evidence for extensive shared polymorphisms at non-MHC immune genes within two genera of North American prairie grouse, *Centrocercus* and *Tympanuchus*. This pattern is primarily attributable to introgression or incomplete lineage sorting following recent divergence and large ancestral effective population size (i.e., weak genetic drift). Our study suggests that prairie grouse may have attained relatively low degree of reciprocal monophyly at nuclear loci. The results also contrast with the patterns of balancing selection we previously found at the MHC of *Centrocercus* and *Tympanuchus* grouse (Minias et al. 2016) and reinforce the rarity of balancing selection in non-MHC immune genes.

## Electronic supplementary material

Fig. S1 (PDF 382 kb)
