# Supplementary material for: Extensive shared polymorphism at non-MHC immune genes in recently diverged North American prairie grouse
Source: Immunogenetics. 2017 Aug 2;70(3):195–204. doi: 10.1007/s00251-017-1024-4 (PMC5818594; doi:10.1007/s00251-017-1024-4)
Supplement: Supplementary file 1 — (PDF 382 kb) [file 251_2017_1024_MOESM1_ESM.pdf]

Fig. S1. Alignments of DNA sequences of ChB6 and IAP-1 exons, TGF-B3 and TRAIL-like introns, and IL-2 promoter of *Centrocercus* (Cemi = *C. minimus*, Ceur = *C. urophasianus*) and *Tympanuchus* (Tycu = *T. cupido*, Typa = *T. pallidicinctu* s, Typh = *T. phasianellus*) grouse. Alleles shared between species are indicated by acronyms given in brackets. Dots indicate the same nucleotide with top sequence. Blue and red shading indicates variable sites within *Tympanuchus* and *Centrocercus* genera, respectively. Codon numeration is indicated above the alignments of exonic regions

ChB6

```
Codons 1–37
Ceur-01 (Cemi)        TGAGAGCATGACATTTGAGTGGCTGCTGAACAATTCTCGGCTGAATGCCCATGAGGCTTGTATTAAGGATGGTGGGAAGAGAGTTCGCCTGGAGAAAACTGTGCCTGGGGAATT
Ceur-02               ......................................................................................A...........................
Ceur-03               ......................................................A...........................................................
Ceur-04               ......................................................A...............................A...........................
Tycu-01 (Typa/Typh)   C..........TG.....................................................................................................
Tycu-02 (Typa/Typh)   C...........G.....................................................................................................
Tycu-03 (Typa/Typh)   C...........G.........................A...........................................................................
Tycu-04 (Typa/Typh)   C.....................................A...........................................................................
Tycu-05               C..........TG.........................A...........................................................................
Tycu-06               C.................................................................................................................
Tycu-07               ...........TG.....................................................................................................
```

IAP-1

```
Codons 1–39
Ceur-01 (Cemi)        TATTTCAGTGGCTCTTTTTCCAGTTTTCCTCAAGACCCGATAACTACTAGGGCTGCCGAAGACCTCTCGCACTTAAGATCTAAGCTTCACAACCCTTCCATGAGTACAGAAGAAGCT
Ceur-02               .....................................................................................................................
Tycu-01 (Typa/Typh)   ......................................A..............................................................................
Tycu-02 (Typa/Typh)   ......................................A..............................................................................
Tycu-03 (Typa/Typh)   ......................................A..............................................................................
Tycu-04 (Typh)        ......................................A.......................................G......................................
Tycu-05               ......................................A..............................................................................
Tycu-06               ......................................A..............................................................................

Codons 40–78
Ceur-01 (Cemi)        AGGCTGCGCACTTTCCATACGTGGCCCCTGATGTTTCTCTCGCCCACCGATCTGGCAAAAGCTGGTCTTTATTACCTGGGGACAGCAGACAAAGTTGCTTGTTTCACCTGTGGTGGT
Ceur-02               .......A.............................................................................................................
Tycu-01 (Typa/Typh)   .....................................................................................................................
Tycu-02 (Typa/Typh)   ...................T.................................................................................................
Tycu-03 (Typa/Typh)   ....................................................................G................................................
Tycu-04 (Typh)        .....................................................................................................................
Tycu-05               ......................................................................................C..............................
Tycu-06               .....................................................................................................................

Codons 79–107
Ceur-01 (Cemi)        CAGTTGAGTAACTGGGAGCCAAAAGATAATGCCATGTCAGAGCATCAGAGGCACTTTCCTAACTGCCCCTTTGTGGAAAACCTCATGCG
Ceur-02               .........................................................................................
Tycu-01 (Typa/Typh)   .........................................................................................
Tycu-02 (Typa/Typh)   .........................................................................................
Tycu-03 (Typa/Typh)   .........................................................................................
Tycu-04 (Typh)        .........................................................................................
Tycu-05               .........................................................................................
Tycu-06               ....................................................................A....................
```

IL-2

```
Ceur-01 (Cemi)      TACCAGTAGACACAGTGGTTAACTTAAACATCTTTAGAACATTTGGTGTGTCAGTAGTATACTGTATGGCATATTCTATGGGGGAGTGGGACAGCCCAAGAGGGAAAAGTGTTTCA
Cemi-01             ..........................................................G.........................................................
Tycu-01 (Typa)      ....................................................................................................................
Tycu-02 (Typa)      C...................................................................................................................
Tycu-03 (Typa)      .........................................................C..........................................................
Tycu-04             ....................................................................................................................
Tycu-05             ....................................................................................................................
Tycu-06             ....................................................................................................................
Tycu-07 (Typa/Typh) ....................................................................................................................
Typa-01             .............................................................................................A......................
Typa-02             C...................................................................................................................
Typh-01             ....................................................................................................................
Typh-02             ....................................................................................................................
Typh-03             .................................................A..................................................................
Typh-04             ...................C................................................................................................

Ceur-01 (Cemi)      TGCAAAAAGCACACTGAGAGAGTGAGTGCCAAAAGTGAGTGTGGGCTTCTCTTACACCAGTGAATGTAGGTAAAATCCCTCTCGCAGTCAAAAAATTCCAAAGTGTCATCAGGGAGG
Cemi-01             .....................................................................................................................
Tycu-01 (Typa)      .....................................................................................................................
Tycu-02 (Typa)      .....................................................................................................................
Tycu-03 (Typa)      .....................................................................................................................
Tycu-04             ....................................A................................................................................
Tycu-05             .........................A...........................................................................................
Tycu-06             .....................................................................................................................
Tycu-07 (Typa/Typh) .....................................................................................................................
Typa-01             .....................................................................................................................
Typa-02             .....................................................................................................................
Typh-01             .....................................................................................................................
Typh-02             .....................A...............................................................................................
Typh-03             .....................................................................................................................
Typh-04             .....................................................................................................................

Ceur-01 (Cemi)      AAAAACAAAAGTAATGATTCTTGCCATACAGGTAAAGCATATGAAAAAATGTGTAATAAAAACCTTCTTTACATGACGCCCCCATCTTTTTCCCCCCAGAGAGAAGAGTATAAATAC
Cemi-01             .....................................................................................................................
Tycu-01 (Typa)      ....................................A................................................................................
Tycu-02 (Typa)      .....................................................................................................................
Tycu-03 (Typa)      .....................................................................................................................
Tycu-04             .....................................................................................................................
Tycu-05             .....................................................................................................................
Tycu-06             .......................................................................................................G.............
Tycu-07 (Typa/Typh) .....................................................................................................................
Typa-01             .....................................................................................................................
Typa-02             .................C...................................................................................................
Typh-01             .....................................................................................................................
Typh-02             .....................................................................................................................
Typh-03             .....................................................................................................................
Typh-04             .....................................................................................................................

Ceur-01 (Cemi)      TAAACAGTCTAATGACAACTTATCACCTCGTATTGTACATCACAATTTGAATACCAGCATA
Cemi-01             .............................................................
Tycu-01 (Typa)      ....................A........................................
Tycu-02 (Typa)      ....................A........................................
Tycu-03 (Typa)      ....................A........................................
Tycu-04             ....................A........................................
Tycu-05             ....................A........................................
Tycu-06             ....................A........................................
Tycu-07 (Typa/Typh) ....................A........................................
Typa-01             ....................A........................................
Typa-02             ....................A........................................
Typh-01             .....................G.......................................
Typh-02             ....................A........................................
Typh-03             ....................A........................................
Typh-04             ....................A........................................
```

TGF-B3

```
Ceur-01 (Cemi)   CTGCTGGGTTTGCATGTTGATCTTACGAGTACACCCACTTCAAACCTCGGTTACAAGTGAGAGAAAAGTGCAGTCTGGGTGGGGTTTGTGTGTGTTTCTGGAAAAGGAATAATTTCA
Ceur-02          .....................................................................................................................
Tycu-01 (Typa)   ....................................................................................................................G
Tycu-02          ......................A.............................................................................................G
Tycu-03          ....................................................................................................................G
Tycu-04          ....................................................................................................................G
Tycu-05          ....................................................................................................................G
Tycu-06          ...................................................................................................................TG
Tycu-10          .............G......................................................................................................G
Typa-01          ....................................................................................................................G
Typh-01          ....................................................................................................................G
Typh-02          ....................................................................................................................G
Typh-03          ....................................................................................................................G
Typh-04          ....................................................................................................................G
Typh-05          ....................................................................................................................G
Typh-06          ....................................................................................................................G
Typh-07          ....................................................................................................................G
Typh-08          ....................................................................................................................G
Typh-09          ....................................................................................................................G

Ceur-01 (Cemi)   TATTTCAGTGTCTCTCTGGGAAGTTCAGCTTGATGGATTCTCAGATTTCCACAGAGGCTCTGCTTTCCAGTTAGGGTGACTGAAACACAAGGAAGTCAGGCATCCTGTGTGAGGTGG
Ceur-02          .....................................................................................................................
Tycu-01 (Typa)   .......................C.............................................................................................
Tycu-02          .......................C.............................................................................................
Tycu-03          .......................C.............................................................................................
Tycu-04          .......................C.............................................................................................
Tycu-05          .......................C.............................................................................................
Tycu-06          .......................C.............................................................................................
Tycu-10          .......................C.............................................................................................
Typa-01          .......................C.............................................................................................
Typh-01          .......................C.............................................................................................
Typh-02          .......................C.............................................................................................
Typh-03          .......................C.............................................................................................
Typh-04          .......................C.............................................................................................
Typh-05          .......................C.............................................................................................
Typh-06          .......................C.............................................................................................
Typh-07          .......................C.............................................................................................
Typh-08          .......................C.................................T...........................................................
Typh-09          .......................C................................C............................................................

Ceur-01 (Cemi)   CCTAGAGAGTCAGTGGCTTGGCTGGCCTAAGACGTAGGAACCTCCTGGCTTGCAGCCCTGGGCTGCAGCCACTGGCCCCCACAGGCTCAATTTCTGAGGCTGTTCTGCAGAGTGCCA
Ceur-02          .....................................................................................................................
Tycu-01 (Typa)   .....................................................................................................................
Tycu-02          .....................................................................................................................
Tycu-03          .....................................................................................................................
Tycu-04          .....................................................................................................................
Tycu-05          ...................................................A.T...............................................................
Tycu-06          .....................................................................................................................
Tycu-10          .....................................................................................................................
Typa-01          ................................T....................................................................................
Typh-01          ...........................................................T.........................................................
Typh-02          ...........................................................T.........................................................
Typh-03          ...........................................................T.........................................................
Typh-04          ...........................................................T.........................................................
Typh-05          ...........................................................T.........................................................
Typh-06          ...........................................................T.........................................................
Typh-07          ...........................................................T.........................................................
Typh-08          ...........................................................T.........................................................
Typh-09          ...........................................................T.........................................................
```

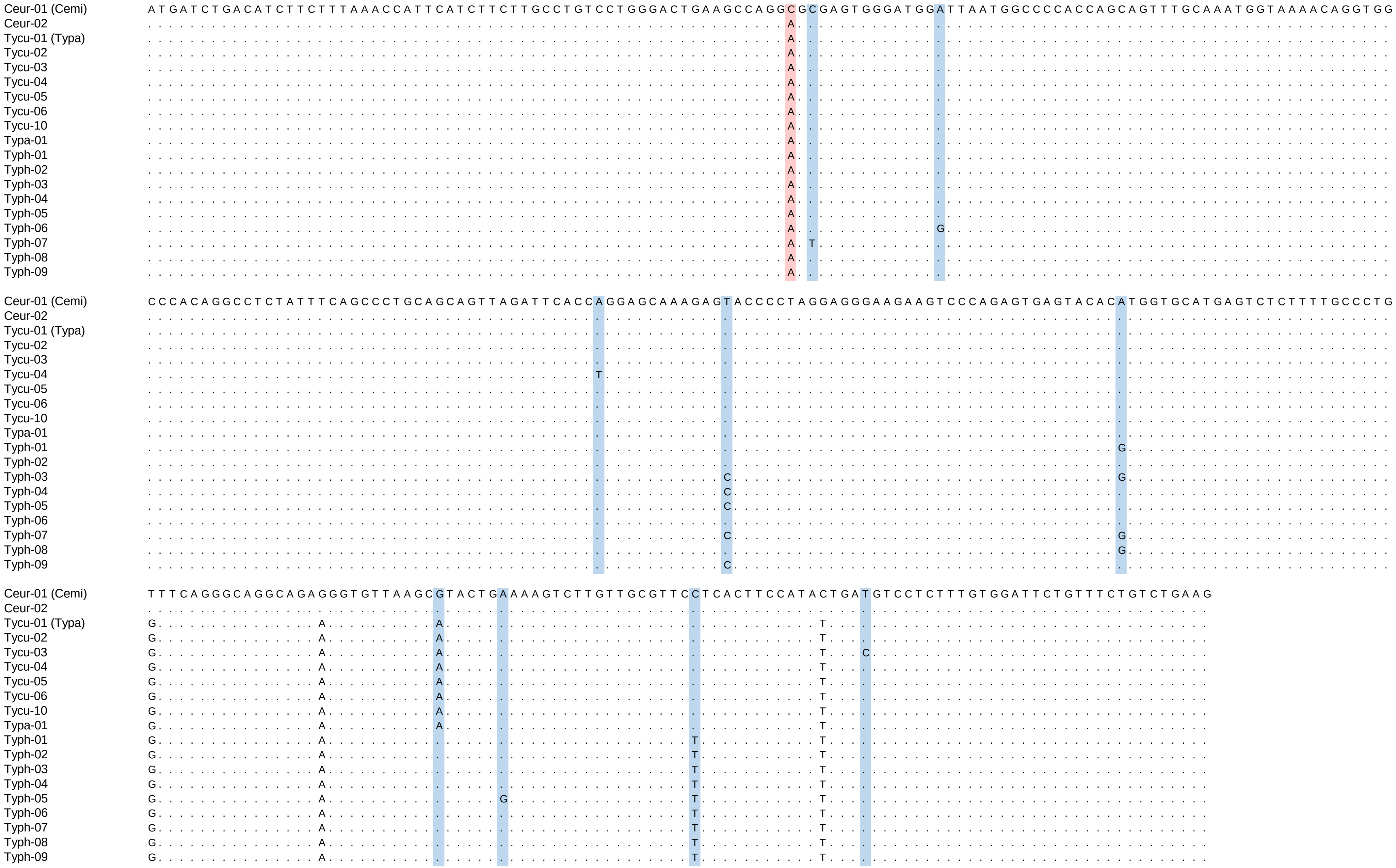
Ceur-01 (Cemi)
Ceur-02
Tycu-01 (Typa)
Tycu-02
Tycu-03
Tycu-04
Tycu-05
Tycu-06
Tycu-10
Typa-01
Typh-01
Typh-02
Typh-03
Typh-04
Typh-05
Typh-06
Typh-07
Typh-08
Typh-09

TRAIL-like

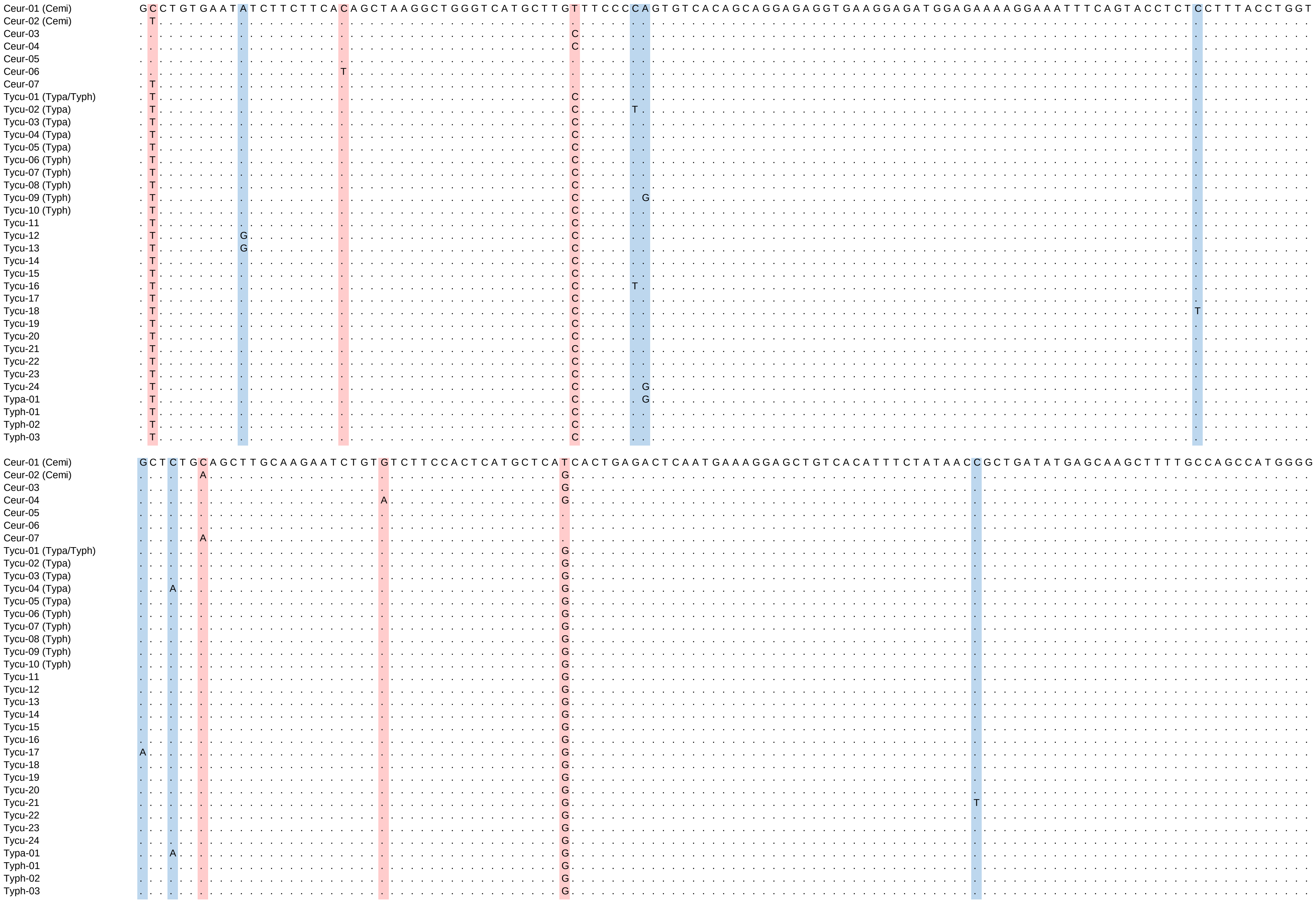
Ceur-01 (Cemi)
Ceur-02 (Cemi)
Ceur-03
Ceur-04
Ceur-05
Ceur-06
Ceur-07
Tycu-01 (Typa/Typh)
Tycu-02 (Typa)
Tycu-03 (Typa)
Tycu-04 (Typa)
Tycu-05 (Typa)
Tycu-06 (Typh)
Tycu-07 (Typh)
Tycu-08 (Typh)
Tycu-09 (Typh)
Tycu-10 (Typh)
Tycu-11
Tycu-12
Tycu-13
Tycu-14
Tycu-15
Tycu-16
Tycu-17
Tycu-18
Tycu-19
Tycu-20
Tycu-21
Tycu-22
Tycu-23
Tycu-24
Typa-01
Typh-01
Typh-02
Typh-03
GCCTGTGAATATCTTCTTCACAGCTAAGGCTGGGTCATGCTTGTTTCCCCAGTGTCACAGCAGGAGAGGTGAAGGAGATGGAGAAAAGGAAATTTCAGTACCTCTCCTTTACCTGGT
GCTCTGCAGCTTGCAAGAATCTGTGTCTTCCACTCATGCTCATCACTGAGACTCAATGAAAGGAGCTGTCACATTTCTATAACCGCTGATATGAGCAAGCTTTTGCCAGCCATGGGG

```
Ceur-01 (Cemi)      ATCATAGGAGGACAAAGAGGATTCAACCCATTCAATTCACAGAACAGGTTTTGGACAGAGCTTGGGACACACTCAGCTTTCTGCCAACTGATTCCTTCCCAGAAGAGCAGCTTCAGT
Ceur-02 (Cemi)      .....................................................................................................................
Ceur-03             .....................................................................................................................
Ceur-04             .....................................................................................................................
Ceur-05             .....................................................................................................................
Ceur-06             .....................................................................................................................
Ceur-07             .....................................................................................................................
Tycu-01 (Typa/Typh) ...........................A...............................A.........................................................
Tycu-02 (Typa)      ...........................A...............................A.........................................................
Tycu-03 (Typa)      ...........................A...............................A.........................................................
Tycu-04 (Typa)      ...........................A...............................A.........................................................
Tycu-05 (Typa)      ...........................A...............................A.........................................................
Tycu-06 (Typh)      ...........................A...............................A...........................T.............................
Tycu-07 (Typh)      ...........................A...............................A...........................T.............................
Tycu-08 (Typh)      ...........................A...............................A.........................................................
Tycu-09 (Typh)      ...........................A...............................A.........................................................
Tycu-10 (Typh)      ...........................A...............................A..A......................................................
Tycu-11             ...........................A...............................A..........G..............................................
Tycu-12             ...........................A...............................A.........................................................
Tycu-13             ...........................A...............................A..........G..............................................
Tycu-14             ...........................A...............................A..........G..............................................
Tycu-15             ...........................A...............................A..........G................T.............................
Tycu-16             ...........................A...............................A.........................................................
Tycu-17             ...........................A...............................A.........................................................
Tycu-18             ...........................A...............................A.........................................................
Tycu-19             ...........................A...............................A.........................................................
Tycu-20             ...........................A...............................A.........................................................
Tycu-21             ...........................A...............................A.........................................................
Tycu-22             ...........................A...............................A..........G..............................................
Tycu-23             ...........................A...............................A.........................................................
Tycu-24             ...........................A...............................A.........................................................
Typa-01             ...........................A...............................A.........................................................
Typh-01             ...........................A...............................A.........................................................
Typh-02             ...........................A...............................A.......................................................T.
Typh-03             ...........................A...............................A..........G................T.............................

Ceur-01 (Cemi)      CCACATTTGTGTCCTGATGGTACCTGCCCATCCTACCATCAGCTTTGTGCCCAGAGAAGGGCTGGGTTATGCTCTATTCCATGGTGTGTCTGTGGGCATCCTGACACAGGAAGAGCC
Ceur-02 (Cemi)      .....................................................................................................................
Ceur-03             .....................................................................................................................
Ceur-04             .....................................................................................................................
Ceur-05             .............G.......................................................................................................
Ceur-06             .....................................................................................................................
Ceur-07             .....................................................................................................................
Tycu-01 (Typa/Typh) ......................G.........................................................................................T....
Tycu-02 (Typa)      ......................G.........................................................................................T....
Tycu-03 (Typa)      ......................G..................................G......................................................T....
Tycu-04 (Typa)      ......................G.........................................................................................T....
Tycu-05 (Typa)      ......................G.........................................................................................T....
Tycu-06 (Typh)      ......................G.........................................................................................T....
Tycu-07 (Typh)      ......................G.........................................................................................T....
Tycu-08 (Typh)      ......................G.........................................................................................T....
Tycu-09 (Typh)      ......................G.........................................................................................T....
Tycu-10 (Typh)      ......................G.........................................................................................T....
Tycu-11             ......................G.........................................................................................T....
Tycu-12             ......................G.........................................................................................T....
Tycu-13             ......................G.........................................................................................T....
Tycu-14             ......................G.........................................................................................T....
Tycu-15             ......................G.........................................................................................T....
Tycu-16             ......................G.....................C...................................................................T....
Tycu-17             ......................G..................................G......................................................T....
Tycu-18             ......................G..................................G......................................................T....
Tycu-19             ......................G..................................G...............................T......................T....
Tycu-20             ......................G............T............................................................................T....
Tycu-21             ......................G.........................................................................................T....
Tycu-22             ......................G.........................................................................................T....
Tycu-23             ......................G.........................................................................................T....
Tycu-24             ......................G......................................................................................T...T....
Typa-01             ......................G.........................................................................................T....
Typh-01             ......................G.......................C.................................................................T....
Typh-02             ......................G.........................................................................................T....
Typh-03             ......................G.........................................................................................T....
```

Ceur-01 (Cemi)	AGACCAAAGGCAGCTGACCTGGGCATGCACCCTGCAGGGATCCCCACTGCGCCCAGCCAAATCATGTGCACTGCCTTACCCTGCTCT
Ceur-02 (Cemi)	....
Ceur-03	....
Ceur-04	....
Ceur-05	....
Ceur-06	....
Ceur-07	....A....................................
Tycu-01 (Typa/Typh)	....
Tycu-02 (Typa)	....
Tycu-03 (Typa)	....
Tycu-04 (Typa)	....
Tycu-05 (Typa)	............T..
Tycu-06 (Typh)	....
Tycu-07 (Typh)	.........A...
Tycu-08 (Typh)	.........A...
Tycu-09 (Typh)	....
Tycu-10 (Typh)	....
Tycu-11	........C..
Tycu-12	....
Tycu-13	....
Tycu-14	....
Tycu-15	....
Tycu-16	....
Tycu-17	....
Tycu-18	....
Tycu-19	....
Tycu-20	....
Tycu-21	....
Tycu-22	.....................................A...
Tycu-23	.................G...
Tycu-24	....
Typa-01	....
Typh-01	....
Typh-02	....
Typh-03	.........A...
